# Comprehensive molecular portrait using next generation sequencing of resected intestinal-type gastric cancer patients dichotomized according to prognosis

**DOI:** 10.1038/srep22982

**Published:** 2016-03-10

**Authors:** E. Bria, S. Pilotto, M. Simbolo, M. Fassan, G. de Manzoni, L. Carbognin, I. Sperduti, M. Brunelli, I. Cataldo, A. Tomezzoli, A. Mafficini, G. Turri, N. Karachaliou, R. Rosell, G. Tortora, A. Scarpa

**Affiliations:** 1 Medical Oncology, Azienda Ospedaliera Universitaria Integrata, University of Verona, Verona, Italy; 2 ARC-NET Applied Research on Cancer Center, University of Verona, Verona, Italy; 3 1^st^ Division of General Surgery, University of Verona, Verona, Italy; 4 Biostatistics, Regina Elena National Cancer Institute, Rome, Italy; 5 Department of Pathology and Diagnostics, University of Verona, Verona, Italy; 6 Pangaea Biotech, Barcelona, Spain; 7 Instituto Oncológico Dr Rosell, Quiron-Dexeus University Hospital, Barcelona, Spain; 8 Catalan Institute of Oncology, Hospital Germans Trias i Pujol, Badalona, Spain; 9 Molecular Oncology Research (MORe) Foundation, Barcelona, Spain; 10 Germans Trias i Pujol Health Sciences Institute and Hospital, Campus Can Ruti, Spain

## Abstract

In this study, we evaluated whether the presence of genetic alterations detected by next generation sequencing may define outcome in a prognostically-selected and histology-restricted population of resected gastric cancer (RGC). Intestinal type RGC samples from 34 patients, including 21 best and 13 worst prognostic performers, were studied. Mutations in 50 cancer-associated genes were evaluated. A significant difference between good and poor prognosis was found according to clinico-pathologic factors. The most commonly mutated genes in the whole population were *PIK3CA* (29.4%), *KRAS* (26.5%)*, TP53* (26.5%) *MET* (8.8%)*, SMAD4* (8.8%) and *STK11* (8.8%). Multiple gene mutations were found in 14/21 (67%) patients with good prognosis, and 3/13 (23%) in the poor prognosis group. A single gene alteration was found in 5/21 (24%) good and 6/13 (46%) poor prognosis patients. No mutation was found in 2/21 (9.5%) and 4/13 (31%) of these groups, respectively. In the overall series, ß-catenin expression was the highest (82.4%), followed by E-Cadherin (76.5%) and FHIT (52.9%). The good prognosis group was characterized by a high mutation rate and microsatellite instability. Our proof-of-principle study demonstrates the feasibility of a molecular profiling approach with the aim to identify potentially druggable pathways and drive the development of customized therapies for RGC.

Gastric cancer (GC) is the fourth most common cancer type and the second cause of cancer deaths^1^. Despite recent progress in the development of new therapeutic approaches, GC prognosis remains poor. Recent molecular analyses suggested that the observed heterogeneity in prognosis and response to treatments should be attributed to the underlying molecular mechanisms driving crucial differences in cancer aggressiveness and treatment outcomes. This heterogeneity manifests through the existence of distinct and clinically meaningful subtypes of GC^2^,^3^. Massive parallel sequencing, also known as next-generation sequencing (NGS), represents an innovative approach to multigene analysis, concurrently screening for multiple potential molecular aberrations thought to drive cancer prognosis and/or guide the choice of therapy^4^,^5^. Here we report on a proof-of-principle study in which we evaluated, in a selected and histology-restricted population (intestinal subtype RGC), whether the presence of specific genetic alterations screened with NGS multigene analysis may further define prognosis. In the context of a preliminary analysis, we proposed and internally validated a prognostic clinical biological risk stratification model based on the combination of clinical and molecular factors. According to this model, the expression of FHIT, APC and HER-2 strongly complement clinical parameters to accurately predict individual patient risk for resected gastric cancer (RGC)^6^.

## Results

### Patient Characteristics

**Figure 1:**
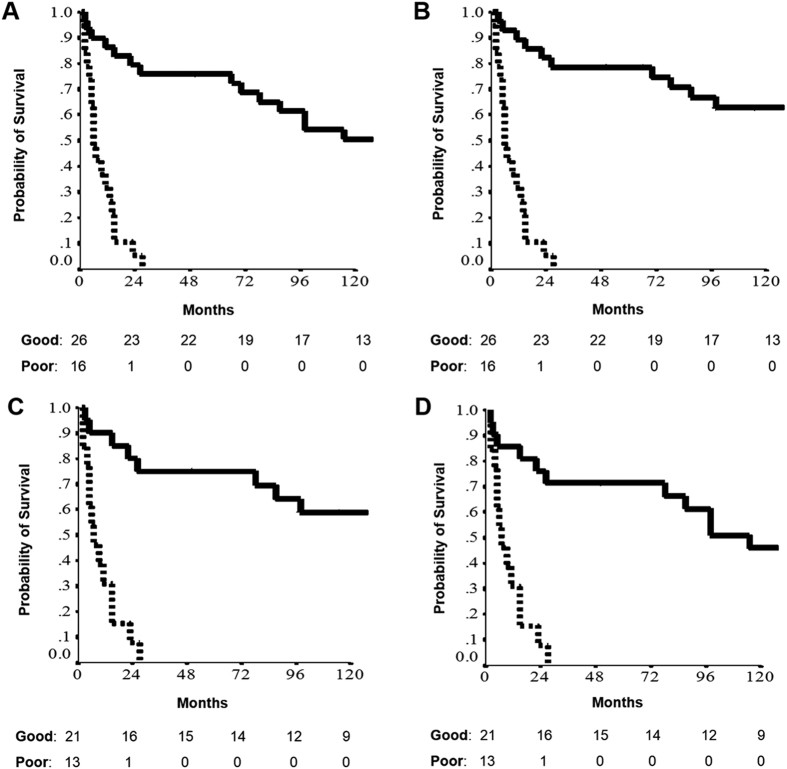
Survival estimates of the 42 patients, being good (26) or poor (16) prognostic performers at cancer specific survival (**A**) and overall survival (**B**) outcomes according to the three-class model^6^. Survival estimates of the 34 patients, being good (21) or poor (13) prognostic performers at both cancer specific survival (**C**) and overall survival (**D**) outcomes.

Forty two patients made up the three-class model, including good (26 patients) and poor (16 patients) prognostic performers. These 42 patients had a 2-year CSS of 82.1% and 5.3%, and a 2-year OS of 79.3% and 5.3%, respectively (Fig. 1A,B). Good and poor prognostic performers had median follow-up of 148 months (range 70–227) and six months (range 5–7), respectively. Among these, 34 patients (21 good and 13 poor performers; attrition rate 80.9%) with intestinal histology (from 114 of the original 208 patients) were considered eligible for the next generation sequencing analysis. Good (21 patients) and poor (13 patients) prognostic performers in the subgroup with intestinal histology had a 2-year CSS of 78.5% and 8.4%, and a 2-year OS of 76.7% and 9.1%, respectively (Fig. 1C,D). Patient characteristics of this group are shown in Table 1. A significant discrepancy between good and poor prognosis was found for median age (p = 0.004), median survival (p < 0.0001), TNM staging (p < 0.0001), tumor size (p = 0.0006), number of resected nodes (p = 0.018), positive node-ratio (p < 0.0001), node status (p = 0.0004), distant metastasis (p = 0.004), site (p = 0.004) and margins (p < 0.0001) (Table 1).

### Molecular features in NGS analysis according to prognosis

**Figure 2:**
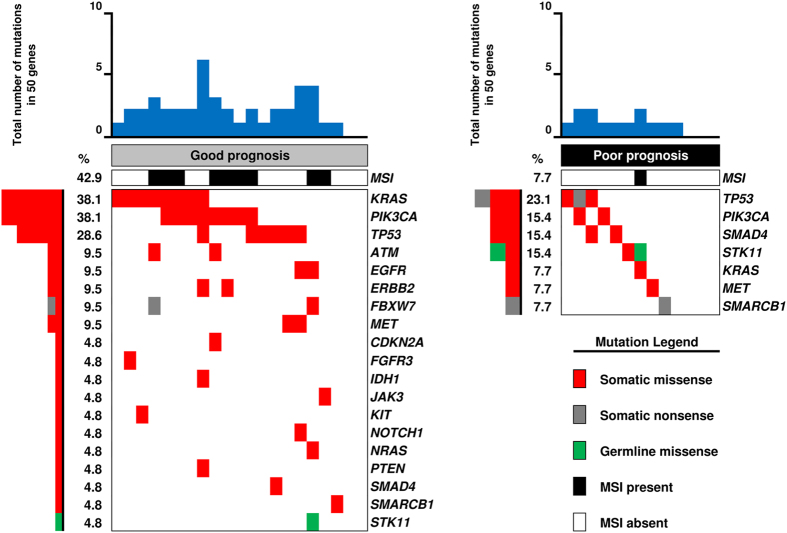
Distribution of mutations in the Next Generation Sequencing analysis according to prognosis. Each column denotes an individual tumor, and each row represents a gene.

**Figure 3:**
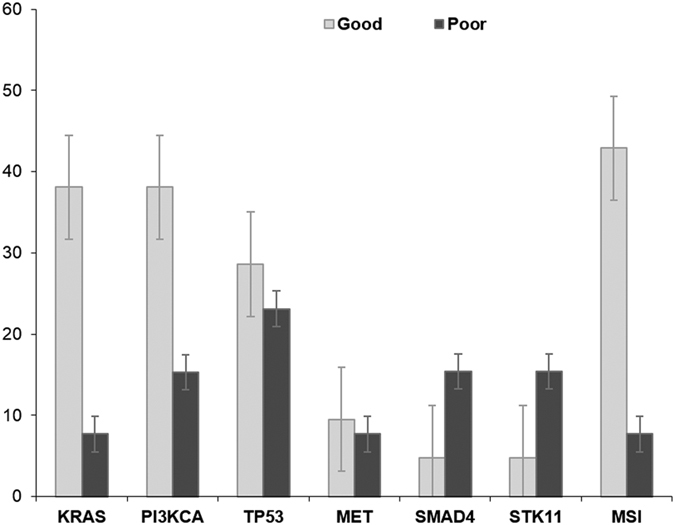
Relative gene frequency (expressed as a percentage) according to prognostic group.

At least one mutation was observed in 28/34 cases (82.4%). Seventeen (50.0%) cases showed concurrent mutations in different genes and six (17.6%) showed no alterations in the 50 genes assayed. The most commonly mutated genes in the whole population were *PIK3CA* (29.4%), *KRAS* (26.5%)*, TP53* (26.5%) *MET* (8.8%)*, SMAD4* (8.8%) and *STK11* (8.8%). Seventeen (Good/Poor: 14/3), 11 (Good/Poor: 5/6) and six (Good/Poor: 2/4) RGCs contained multiple, single or no gene alteration, respectively. *ATM*, *CDKN2A*, *EGFR*, *ERBB2*, *FBXW7* and *SMARCB1* were mutated in two cases, and *FGFR3*, *IDH1*, *JAK3*, *KIT*, *NOTCH1*, *NRAS* and *PTEN* in one. Distribution of patients’ molecular characteristics in the NGS analysis according to prognosis is shown in Figs 2 and 3. The most frequently mutated genes are shown in Table 2. The good prognosis group was characterized by a high mutation rate with at least one mutation observed in 19 (90.5%) cases. In this group only two cases (9.5%) did not present any alteration, while 14 cases (66.7%) showed two or more concurrent mutations. *KRAS* and *PIK3CA* represented the most frequently altered genes in the good prognosis group (38.1%). *TP53* gene was the second most frequently altered gene in the group (28.6%). Of interest was the fact that the good prognosis patients showed alterations in several genes involved in targetable pathways. In this regard, genes belonging to the mTOR pathway (such as *FBXW7*, *PIK3CA* and *PTEN*) were altered in 10 cases (47.6%). Tyrosine kinase receptors (*EGFR*, *ERBB2*, *FGFR3*, *KDR*, *KIT* and *MET*) were altered in seven cases (33.3%). Patients in the good prognosis group were characterized by microsatellite instability (MSI) in 42.9% of cases. The poor prognosis group was characterized by a relative low mutation rate compared with good prognosis patients. Four (30.8%) cases did not present any alteration in the 50 genes assayed, six (46.2%) cases showed only one mutation and two concurrent mutations were observed only in three (23.1%) cases. *TP53* was the most frequently altered gene (23.1%). *PIK3CA*, *SMAD4* and *STK11* were altered in two samples each (15.4%). A *KRAS* alteration was observed only in one case (7.7%). The low mutation rate and the small number of samples made it more difficult to identify a targetable pathway, as was the case in the good prognosis group. The mTOR pathway was altered in two cases (15.4%) and one case showed alteration in the tyrosine kinase receptor MET (7.7%). Microsatellite instability was observed only in one patient with poor prognosis (7.7%).

### Expression features by IHC and FISH according to prognosis

The results of IHC and FISH analyses are summarized in Table 3 and Supplementary Table 2. We investigated the expression of the following proteins: beta-catenin; FHIT, E-Cadherin, APC, CDX2, MET, TOPO2A, HER2 and p53. In the overall series, beta-catenin was most highly expressed (82.4%), followed by E-Cadherin (76.5%) and FHIT (52.9%). According to prognosis, beta-catenin was expressed in 76.2% and 92.3% of the good and bad prognosis groups, respectively (p = 0.37). E-Cadherin expression was high in 66.6% of the good prognosis patients, compared to 92.3% of the bad prognosis group (p = 0.12). A statistically significant difference according to prognosis was observed for FHIT. In fact positive cases were identified in 71.4% of the good prognosis subgroup and only in 23.1% of the bad prognosis group (p = 0.012). FISH analysis demonstrated amplification of TOPO2A in 35.3% of cases, MET in 20.6% and HER2 in 5.9% of cases. No statistically significant correlation between FISH analysis and prognosis was observed.

## Discussion

In this study we analyzed genetic alterations using NGS and protein expression by IHC and FISH according to the different prognoses of intestinal-type RGC. Although no specific genes associated with prognosis were identified, a specific molecular profile emerged as a tool to predict and define clinical outcomes.

The concept to molecularly characterized different prognostic groups in order to identify the potential existence of differential driver molecular alterations is innovative and promising. In this regard, the tumor genetic analysis of the LUX-Lung 8, a phase III trial of afatinib versus erlotinib in squamous cell carcinoma of the lung, was performed in 2 subgroups of patients distinct according to treatment response^7^.

In recent years, efforts have been made to apply NGS technologies to clinical practice, developing innovative predictive models for GC^8^,^9^. Among them, the TGCA Research Network and ACRG classifications have integrated the results of a wide scale molecular analysis into two different and partially overlapping models encompassing four molecular subtypes^3^,^10^. Similar to the results of these pivotal molecular studies, we demonstrated that patients with good prognosis are characterized by a hypermutated profile (at least one mutation observed in 90.5% cases; only 9.5% did not present any alteration) compared with the poor prognosis subgroup (at least one mutation observed in 46.2% cases; 30.8% had no alteration). The median mutation rate (number of total mutations divided by total number of cases) in the good prognosis group was two per sample, whereas in the poor prognosis group this was 0.9 per sample. Moreover, the good prognosis subgroup showed MSI in 42.9% of cases, compared to 7.7% in the poor prognosis subgroup. To date, MSI positivity has generally been associated with intestinal histology^3^. However, our analysis supports the fact that prognosis, more than histological subtype, may suggest the presence of this underlying molecular feature.

The most commonly mutated genes in our population were *PIK3CA* (29.4%), *KRAS* (26.5%) and *TP53* (26.5%). *PIK3CA* mutations were highly represented in our good prognostic subgroup (38.1%) and this evidence is coherent with the available data, supporting the stronger impact of the PIK pathway in MSI positive tumors characterized by intestinal histology^3^. Similarly, the proportion of *KRAS* mutant patients was relatively high in the good prognosis population (38.1% compared to 7.7% in the poor prognosis group) suggesting that hyperactivation of receptor tyrosine kinase signaling positively impacts on prognosis. In this regard, the good prognosis subgroup had more alterations in the mTOR pathway (47.6%) and tyrosine kinase receptors (33.3%) compared to the poor prognosis group (altered in 15.4% and 7.7% of cases, respectively). *TP53* mutations are equally stratified in the two prognostic populations according to that described by Cristescu *et al.* where *TP53* status reflects intermediate prognosis^3^.

As suggested in our preliminary study^6^, IHC and FISH analyses confirmed the potential predictive role of FHIT (p = 0.012) and APC (p = 0.15) expression as positive prognostic biomarkers for RGC. Moreover, the expression of CDX2 and TOPO2A seem to correlate with favorable prognosis. The validated predictive biomarker HER-2 is expressed at low frequency in our series (4.8% and 7.7% of good and poor prognosis patients, respectively), however this is in close agreement with that reported in other studies^11^. Regarding MET amplification, although its predictive role is widely recognized, available results regarding its prognostic significance are inconclusive. Moreover, several issues regarding tissue sampling, detection methods and cutoff values need to be clarified before definitive conclusions can be drawn^12^.

Recently, a comprehensive analysis of NGS and IHC in advanced GC demonstrated the existence of a complicated arrangement of protein expression and gene alterations, supporting the overall results of our study in RGC^10^. Although the small sample size does not allow us to draw definitive conclusions, our analysis represents an innovative concept in the application of molecular profiling techniques, potentially leading to better allocation of resources. The preliminary stratification of patients according to prognosis allows identification of those patients emerging as ‘outliers’, or rather biologically different from the majority of the population affected by the same morphologically-defined disease. These outlier patients harbor some specific molecular aberrations that explain their unusual degree or duration of clinical benefit derived from an otherwise relatively ineffective treatment^13^. In this regard, curiosity about these ‘exceptional’ responders has historically driven the identification and validation of specific genomic alterations that can impact on patient prognosis and determine susceptibility to selective targeted therapies, thus changing the natural history of some subgroups of cancer patients.

Regarding the future perspective for the presented analysis, an external validation of the identified molecular differences between the two prognostic subgroups will be performed. Moreover, considering the emerging clinical application of liquid biopsy as a feasible, non-invasive method for detecting and monitoring genomic alterations, this approach might represent a future step of the project, in the context of a prospective cohort of patients.

The clinical application of molecular tumor profiling assays, such as NGS technologies, able to codify complicated factors such as tumor heterogeneity should be investigated in larger samples in order to complement clinical parameters to accurately predict individual patient risk and possibility of response in the context of clinical trials. In this context, our analysis represents a proof-of-principle study that demonstrates the feasibility of a comprehensive molecular profiling approach for cancer with the aim to identify potentially druggable pathways. This preliminary step is essential in paving the way for ideation of innovative and biologically relevant clinical trials (such as basket and umbrella studies) that rigorously test the efficacy of molecular targeted agents assigned on the basis of a specific genomic profile.

## Methods

### Patients

Based on the results of a previously published three-class model (prognostic accuracy for overall survival AUC 0.821, standard error 0.03), 34 tumor blocks from an original sample (208 patients) were included from those patients who had the best and the worst prognosis, both in terms of cancer-specific survival (CSS), and overall survival (OS)^6^. Only patients with intestinal histology were considered eligible for the current analysis in order to have a homogeneous population. A formalin-fixed paraffin-embedded (FFPE) tumor excision was available and analyzed for mutations in 50 genes with deep sequencing technology. We confirm that all the experiments were performed in accordance with relevant guidelines and regulations and that all the experimental protocols were approved by the local Ethics Committee of the Azienda Ospedaliera Universitaria Integrata of Verona (protocol 19147 of the 28/11/2011). The informed consent was obtained from all the subjects included in the study.

### NGS of multiplex PCR amplicons

Four 10-μm paraffin sections were manually microdissected to ensure that each tumor sample contained at least 70% of neoplastic cells. DNA was purified and its quality assessed as described^14^. Deep sequencing was performed using the Ion Torrent platform (Life Technologies) following established protocols^15^. For multiplex PCR amplification, 40 ng DNA was used, using the Ion AmpliSeq Cancer Hotspot Panel v2 (Life Technologies) that explores selected regions of the following 50 cancer-associated genes (in alphabetical order):* ABL1, AKT1, ALK, APC, ATM, BRAF, CDH1, CDKN2A, CSF1R, CTNNB1, EGFR, ERBB2, ERBB4, EZH2, FBXW7, FGFR1, FGFR2, FGFR3, FLT3, GNA11, GNAS, GNAQ, HNF1A, HRAS, JAK2, JAK3, IDH1, IDH2, KDR/VEGFR2, KIT, KRAS, MET, MLH1, MPL, NOTCH1, NPM1, NRAS, PDGFRA, PIK3CA, PTEN, PTPN11, RB1, RET, SMAD4, SMARCB1, SMO, SRC, STK11, TP53, VHL*. Emulsion PCR was performed with the OneTouch OT2 system (Life Technologies). The quality of the obtained library was evaluated by High Sensitivity Assay on the Agilent 2100 Bioanalyzer on-chip electrophoresis (Agilent Technologies). Sequencing was run on the Ion Torrent PGM (Life Technologies) loaded with a 316 chip. Data analysis, including alignment to the hg19 human reference genome and variant calling, utilized the Torrent Suite Software ver. 3.6 (Life Technologies). Filtered variants were annotated using the SnpEff software ver. 3.1 (alignments were visually verified with the Integrative Genomics Viewer; IGV ver. 2.1, Broad Institute).

### DNA Sanger Sequencing

To validate NGS results, KRAS (exons 2, 3), and TP53 (exons 5, 6, 7, 8) specific PCR fragments were analyzed by Sanger sequencing. PCR products were purified using Agencourt AMPure XP magnetic beads (Beckman Coulter), labeled with Big Dye terminator v3.1 (Applied Biosystems). Agencourt CleanSEQ magnetic beads (Beckman Coulter) were used for post-labeling purification. Sequence analysis was performed on an Applied Biosystems 3130xl Genetic Analyser.

### IHC staining and FISH analysis

FFPE tissues of cases of GC with intestinal histology were used to construct tissue microarrays (TMAs) as previously described^16^. Each samples was represented by three cores of 1 mm of diameter. TMAs were immunostained using the antibodies listed in Supplementary Table S1. Detection was carried out using Dako EnVision Plus-HRP kit (Dako). Slides were scanned with ScanScope® GL System (Aperio Technologies, Vista, CA) and visualized using ImageScope™ Software (Aperio Technologies). Immunoreactivity evaluation for each antigen is detailed in the Appendix (Methods section). FISH was carried out using HER2, MET and TOPO2A locus-specific and chromosome 17 centromeric probes (Vysis, Downers Grove, IL) diluted 1:100 in tDenHyb1 buffer (Insitus, Albuquerque, MN). The slides were examined using an Olympus BX61 (Bremerhaven, Germany) and the appropriate filters. Annotation of tumors was carried out as previously described^17^,^18^.

### Statistical analysis

Descriptive statistics was used to summarize pertinent study information. Follow-up was analysed and reported according to Shuster^19^. The correlation between variables were analysed according to chi-square, Student’s t, and Mann-Whitney (nonparametric) tests. CSS and OS were calculated by the Kaplan–Meier product limit method from the date of surgery until death due to any cause. The Log-rank analysis was adopted to assess differences between curves. Significance was defined at the p < 0.05 level. The SPSS® (18.0) licensed statistical program was used for all analyses.

## Additional Information

**How to cite this article**: Bria, E. *et al.* Comprehensive molecular portrait using next generation sequencing of resected intestinal-type gastric cancer patients dichotomized according to prognosis. *Sci. Rep.* **6**, 22982; doi: 10.1038/srep22982 (2016).

## Supplementary Material

Supplementary Information

## Figures and Tables

**Table 1:** Clinical and pathological characteristics of the 34 patients evaluable for Next Generation Sequencing analysis.

	Good prognosis (n = 21)	Poor prognosis (n = 13)	*p-value*
	Patient number (%)		
Median age [years]	64.4	74.5	*0.004*
* *Range	[47–88]	[63–82]	
Median survival [months]	98	7	<*0.0001*
* *Range	[2–232]	[2–27]	
Gender			
Male	13 (61.8)	9 (69.2)	*0.727*
Female	8 (38.2)	4 (30.8)	
TNM Staging [according to TNM 7th edition]			
I–II	20 (95,2)	0 (0.0)	<*0.0001* [I–II *vs* III–IV]
III–IV	1 (4.8)	13 (100.0)	
Tumor size [T descriptor according to TNM 7th edition]			
1–2–3	21 (100.0)	7 (53.8)	*0.0006* [1–3 *vs* 4]
4	0 (0.0)	6 (46.2)	
Median resected lymph nodes	45	29	*0.018*
* *Range	[6–108]	[5–48]	
Positive node ratio			
<10%	18 (85.7)	2 (15.3)	<*0.0001*
≥10%	3 (14.3)	11 (84.7)	
Node status [N descriptor according to TNM 7th edition]			
0–1–2	21 (100.0)	9 (69.2)	*0.0004* [0–2 *vs* 3]
3	0 (0.0)	4 (30.8)	
Distant metastasis [M descriptor according to TNM 7th edition]			
M0	21 (100.0)	8 (61.5)	*0.004*
M1	0 (0.0)	5 (38.5)	
Site			
Antrum/Body	19 (85.7)	5 (38.5)	*0.004*
Fundus	2 (14.3)	8 (61.5)	
Margins			
R0	20 (95.2)	5 (38.5)	<*0.0001* [0 *vs* 1–2]
R1–R2	1 (4.8)	8 (61.5)	
Grading			
1–2	10 (47.6)	6 (46.2)	*0.452* [1–2 *vs* 3]
3	11 (52.4)	7 (53.8)	

The differences observed in term of clinicopathological features between good and poor prognosis patients are reported with the corresponding *p-value*.

**Table 2:** Molecular characteristics of the 34 patients (overall number and rate) in the Next Generation Sequencing analysis (only those genes mutated in more than three cases are reported).

Gene mutation	Good prognosis (n = 21)	Poor prognosis (n = 13)	*p-value*
	Patients number (%; 95% CI)		
KRAS			
No	13 (61.9; 41.1–82.6)	12 (92.3; 77.8–99.3)	*0.11*
Yes	8 (38.1; 17.3–58.8)	1 (7.7; 1.0–22.1)	
PI3KCA			
No	13 (61.9; 41.1–82.6)	11 (84.6; 65.0–99.4)	*0.25*
Yes	8 (38.1; 17.3–58.8)	2 (15.4; 1.0–34.9)	
TP53			
No	15 (71.4; 52.1–90.7)	10 (76.9; 54.0–99.8)	*0.99*
Yes	6 (28.6; 9.2–47.8)	3 (23.1; 1.0–45.9)	
MET			
No	19 (90.5; 77.9–99.0)	12 (92.3; 77.8–99.8)	*0.99*
Yes	2 (9.5; 1.0–22.0)	1 (7.7; 1.0–22.1)	
SMAD4			
No	20 (95.2; 86.1–99.0)	11 (84.6; 65.0–99.5)	*0.54*
Yes	1 (4.8; 1.0–13.8)	2 (15.4; 1.0–34.9)	
STK11			
No	20 (95.2; 86.1–99.6)	11 (84.6; 65.0–99.7)	*0.54*
Yes	1 (4.8; 1.0–13.8)	2 (15.4; 1.0–34.9)	
MSI			
Absent	12 (57.1; 35.9–78.3)	12 (92.3; 77.8–99.0)	*0.51*
Present	9 (42.9; 21.6–64.0)	1 (7.7; 1.0–22.1)	

The differences observed in term of molecular features between good and poor prognosis patients are reported with the corresponding *p-value*.

**Table 3:** Immunohistochemical and fluorescence *in situ* hybridization (FISH) analyses of the 34 patients evaluable for Next Generation Sequencing.

	Good prognosis (n = 21)	Poor prognosis (n = 13)	*p-value*
	Patients number (%)		
Beta-Catenin			
Negative	5 (23.8)	1 (7.7)	*0.37*
Intermediate/Positive	16 (76.2)	12 (92.3)	
FHIT			
Negative	6 (28.6)	10 (76.9)	*0.012*
Positive	15 (71.4)	3 (23.1)	
E-Cadherin			
Negative	7 (33.3)	1 (7.7)	*0.12*
Positive	14 (66.6)	12 (92.3)	
APC			
Negative	10 (50.0)	10 (76.9)	*0.15*
Positive	10 (50.0)	3 (23.1)	
CDX2			
Negative	9 (42.8)	10 (76.9)	*0.08*
Positive	12 (57.2)	3 (23.1)	
MET			
Amplified	4 (19.0)	3 (23.0)	*1.0*
Non-amplified	17 (81.0)	10 (77.0)	
TOPO2A			
Amplified	5 (23.8)	7 (53.8)	*0.14*
Non-amplified	16 (76.2)	6 (46.2)	
HER2			
Amplified	1 (4.8)	1 (7.7)	*1.0*
Non-amplified	20 (95.2)	12 (92.3)	
p53			
Weak	14 (66.6)	8 (61.5)	*1.0*
Strong	7 (33.3)	5 (38.5)	

The discrepancies observed in term of pathological features between good and poor prognosis patients are reported with the corresponding *p-value*.
