# Supplementary material for: Comprehensive molecular portrait using next generation sequencing of resected intestinal-type gastric cancer patients dichotomized according to prognosis
Source: Sci Rep. 2016 Mar 10;6:22982. doi: 10.1038/srep22982 (PMC4785368; doi:10.1038/srep22982)
Supplement: Supplementary Information [file srep22982-s1.pdf]

Title:

Comprehensive molecular portrait using next generation sequencing of resected intestinal-type gastric cancer patients dichotomized according to prognosis.

Authors:

E. Bria[1*], S. Pilotto[1*], M. Simbolo[2], M. Fassan[2], G. de Manzoni[3], L. Carbognin[1], I. Sperduti[4], M. Brunelli[5], I. Cataldo[5], A. Tomezzoli[5], A. Mafficini[5], G. Turri[5], N. Karachaliou[6], R. Rosell[6,7,8,9,10], G. Tortora[1#], A. Scarpa[2,5#].

*E.B. and S.P. share the first co-authorship.

#G.T. and A.S. share the last co-authorship.

Supplementary Table 1. Antibodies used for immunohistochemical analyses.

**Antibody**	**Clone/code number**	**Manufacturer[a]**
APC	Code Ab120	*Abcam (Cambridge, UK)*
ß-Catenin	Clone 15-B8	*Sigma-Aldrich (St Louis,MO)*
E-Cadherin	Clone NCH-38	*Dako (Carpinteria,CA)*
Mlh1	Clone 168-15	*Pharmingen (San Diego, CA)*
Msh2	Clone G129-1129	*Pharmingen (San Diego, CA)*
Msh6	Clone 44	*Pharmingen (San Diego, CA)*
FHIT	Code 719000	*Zymed Lab (San Francisco,CA)*
CDX2	CDX2-88	*Biogenex (Fremont,CA)*
p53	Clone DO7	*Dako (Carpinteria,CA)*
p21	Clone SX118	*Dako (Carpinteria,CA)*
Her2	Code K5207	*Dako (Carpinteria,CA)*

[a] Antigen detection was performed according to manufacturers' instructions.

Supplementary Table 2. *MET* characteristics of the 34 patients included in the FISH analysis.

MET according to FISH analysis	Good prognosis (n = 21)	Poor prognosis (n = 13)
	Patients number (%)	
Gene copy number (GCN)		
Gain (≥ 5)	7 (33.3)	5 (38.4)
No gain (< 5)	14 (66.6)	8 (61.5)
Chromosomal alterations		
Monosomy	3 (14.3)	3 (23.1)
Trisomy	1 (4.7)	0 (0.0)
None	17 (81.0)	10 (76.9)

Legend - Table S2. GCN: gene copy number.

METHODS

IHC details. APC immunostaining was detected in the cytoplasm of normal gastric mucosa. Tumors were considered as bearing APC alterations when there was a complete absence of staining for APC in all neoplastic cells *(Davies ML et al. 2007. Brit J Cancer 97:384-390)*. β-catenin and E-cadherin expression was predominantly observed at the cell membrane of normal gastric cells. β-catenin expression was scored as altered when ›10% of the tumor cells showed nuclear and/or cytoplasmic immunoreactivity (*Lim S et al. 2003. Histopathology 42:128-136*). Loss of membranous expression of E-cadherin was defined as either no immunoreactivity or less than 10% membranous positive tumor cells (*Lim S et al. 2003. Histopathology 42:128-136*). Lack of expression of MLH1, MSH2, MSH6 proteins was defined as complete absence of nuclear staining in tumor cells. Intact nuclear staining of the peritumoral normal mucosa cells and lymphocytes served as internal positive control and was required for adequate evaluation (*Losi L et al. 2005. Am J Gastroenterol 100:2280-2287*). Cases were considered positive for FHIT staining if at least one tissue core showed cytoplasmic staining and if 10% or more of the neoplastic cells were stained (*Capuzzi D et al. 2000. Cancer 88:24–34*). The evidence of cytoplasmic staining of adjacent non-neoplastic epithelial fundic glands served as internal positive controls. Nuclear staining of CDX2 was considered and cases displaying more than 10% immunopositive cells were scored as positive. The expression of p53 and its downstream effector p21 was evaluated semi-quantitatively by estimating the fraction of cells positive for nuclear staining. Tumors were considered to harbor *P53* alterations when two conditions were present: (i) immunostaining in more than 50% of neoplastic cells and (ii) a ratio of more than two between the fraction of p53 and p21 positive cells (*Chilosi M et al. 1996. Blood 88:4012-4020*). HER2 evaluation was performed in agreement with the recommendations from the manufacturer of the diagnostic HercepTest kit (Hoffman's criteria) (*American Society of Clinical Oncology-College of American Pathologists Guideline Recommendations for Human Epidermal Growth Factor Receptor 2 Testing in Breast Cancer. 2007. J Clin Oncol 25:118-145*). Three independent observers scored protein expression.

FISH details. FISH was performed by using the PathVysion Her2⁄neu DNA probe and the topoisomerase-IIa kit from Abbott⁄Vysis Inc, Milan, Italy. The technical procedure was performed according to the manufacturers' instructions. The slides were examined using an Olympus BX61, (Bremerhaven, Germany) with appropriate filters for Spectrum Orange (locus specific probe Her2 or topoisomerase-IIa; Abbott), Spectrum-Green (centromeric probe 17; Abbott) and the UV Filter for the DAPI nuclear counterstain. The signals were recorded with a CCD camera (Olympus). Specimens were recorded as amplified if the ratio

of Her2 ⁄ neu signals to chromosome 17 centromere signals was higher than 2.2, according to the ASCO⁄CAP score (*Brunelli M et al. Am J Clin Pathol 2008. 129:907–911; Lal P et al. Am J Clin Pathol 2004. 121:631–636*). Specimens were considered to be amplified for topoisomerase-IIa if the ratio of topoisomerase-IIa signals to chromosome 17 centromere signals was >2. Deletion of topoisomerase-IIa was noted when a ratio of <0.8 (topoisomerase-IIa ⁄ CEP17) was observed in >30% of neoplastic nuclei (*Brunello E et al. Histopathology 2012. 60:482–488*).
